# Echocardiographic determination of right ventricular volumes and ejection fraction: Validation of a truncated cone and rhomboid pyramid formula

**DOI:** 10.1371/journal.pone.0290418

**Published:** 2023-08-18

**Authors:** Ghazi Al Ateah, Annemarie Kirschfink, Michael Frick, Mohammad Almalla, Michael Becker, Christian Cornelissen, Rainer Hoffmann, Nikolaus Marx, Ertunc Altiok

**Affiliations:** 1 Department of Cardiology, Nephrology and Internal Intensive Care Medicine, Rhein-Maas Klinikum, Wuerselen, Germany; 2 Department of Cardiology, Angiology and Intensive Care, University Hospital RWTH Aachen, Aachen, Germany; 3 Department of Pneumology and Intensive Care, University Hospital RWTH Aachen, Aachen, Germany; 4 Department of Cardiology, St. Bonifatius Hospital, Lingen, Germany; Oswaldo Cruz Foundation, BRAZIL

## Abstract

**Background:**

Echocardiographic assessment of right ventricular (RV) measurements may be challenging. The aim of this study was to develop a formula for calculation of RV volumes and function based on measurements of linear dimensions by 2-dimensional (2D) transthoracic echocardiography (TTE) in comparison to cardiovascular magnetic resonance (CMR).

**Methods:**

129 consecutive patients with standard TTE and RV analysis by CMR were included. A formula based on the geometric assumptions of a truncated cone minus a truncated rhomboid pyramid was developed for calculations of RV end-diastolic volume (EDV) and RV end-systolic volume (ESV) by using the basal diameter of the RV (Dd and Ds) and the baso-apical length (Ld and Ls) in apical 4-chamber TTE views: RV EDV = 1.21 * Dd^2^ * Ld, and RV ESV = 1.21 * Ds^2^ * Ls.

**Results:**

Calculations of RV EDV (ΔRV EDV = 10.2±26.4 ml to CMR, r = 0.889), RV ESV (ΔRV ESV = 4.5±18.4 ml to CMR, r = 0.921) and RV EF (ΔRV EF = 0.5±4.0% to CMR, r = 0.905) with the cone-pyramid formula (CPF) highly agreed with CMR. Impaired RV function on CMR (n = 52) was identified with a trend to higher accuracy by CPF than by conventional echocardiographic parameters (tricuspid annular plane systolic excursion (TAPSE) and fractional area change (FAC)).

**Conclusion:**

Calculations of RV volumes and RV function by 2D TTE with the newly developed CPF were in high concordance to measurements by CMR. Accuracy for detection of patients with reduced RV function were higher by the proposed 2D TTE CPF method than by conventional echocardiographic parameters of TAPSE and RV FAC.

## Introduction

The right ventricle (RV) has been considered to be no more than a conduit and reservoir for a long time. Meanwhile, the importance of the RV in both acquired and congenital heart disease has been recognized. RV function has an important impact on patient outcome in various types of congenital and acquired heart disease. Echocardiographic assessment of the RV is challenging. 2-dimensional (2D) approaches for assessment of size and function are limited in providing accurate results, predominantly due to the complex anatomy of the RV [1]. The RV is composed of 3 distinct portions: the smooth muscular inflow, the trabecular apical region, and the outflow tract. Established 2D echocardiographic parameters of RV size are linear dimensions measured in parasternal and apical views as well as end-diastolic and end-systolic areas assessed in apical 4-chamber view. Proposed 2D transthoracic echocardiography (TTE) variables of RV function are tricuspid annular plane systolic excursion (TAPSE), RV fractional area change (FAC) measured in apical 4-chamber view, tricuspid annular systolic velocity (S´) measured by tissue Doppler, myocardial performance index (MPI) by pulsed-wave Doppler or tissue Doppler and strain-analysis of the RV free wall by speckle-tracking echocardiography [2, 3]. 3-dimensional (3D) TTE may overcome the geometrical limitations of 2D echocardiography. However, 3D echocardiographic assessment of the RV is technically challenging and not applicable in a relevant number of patients with impaired image quality. Due to these limitations, it has been proposed to be used only in laboratories with appropriate 3D platforms and experience [3]. Hence, there is a need for a 2D echocardiography-based method providing accurate RV volumetric analysis.

This study sought to evaluate a formula for calculation of RV volumes and function developed on the basis of geometric assumptions using only two linear dimensions of conventional apical 4-chamber views obtained by 2D TTE and to compare these calculations with measurements performed by cardiac magnetic resonance (CMR) as the reference method [4].

## Methods

In this single center study 129 consecutive patients (50±18 years; 89 male) who were scheduled for RV analysis by CMR for various indications and in whom concomitant standard 2D TTE was performed were included. Patients not eligible for CMR (e.g. patients with pacemakers, defibrillators, claustrophobia) were excluded. This study was approved and the need for consent was waived by the local ethics committee (EK 012/21). The data was recorded on a personal basis and pseudonymised prior to evaluation. The collected data was not passed on outside the department.

### TTE protocol

Echocardiograms were performed with a GE Vivid E9 system (GE Healthcare, Ultrasound, Horton, Norway, 2D transthoracic probe M5S) or a Philips IE33 system (Philips Medical Systems, Andover, Massachusetts, USA, 2D transthoracic probe S5-1). Parasternal long- and short axis views as well as 3 apical views (4-chamber, 2-chamber and long-axis) were acquired with typical frame rates >50 frames/s. Care was taken to acquire apical 4-chamber views without left ventricular (LV) apex shortening with rotating the transducer until the maximal planes were obtained with both ventricles as wide as possible without focusing on a specific ventricle. Additionally, interventricular septum and interatrial septum were aligned with each other and perpendicular to the horizontal planes. Standard echocardiographic measurements were performed including assessment of LV ejection fraction and systolic pulmonary artery pressure by continuous-wave Doppler. TAPSE was assessed by placing the M-mode line at the lateral tricuspid valve annulus in the apical 4-chamber view by measuring the extent of the annulus movement during systole. In the same view RV area was measured at end-diastole and end-systole. RV FAC was calculated as: RV FAC = (RV diastolic area–RV systolic area) / RV diastolic area * 100.

**Fig 1:**
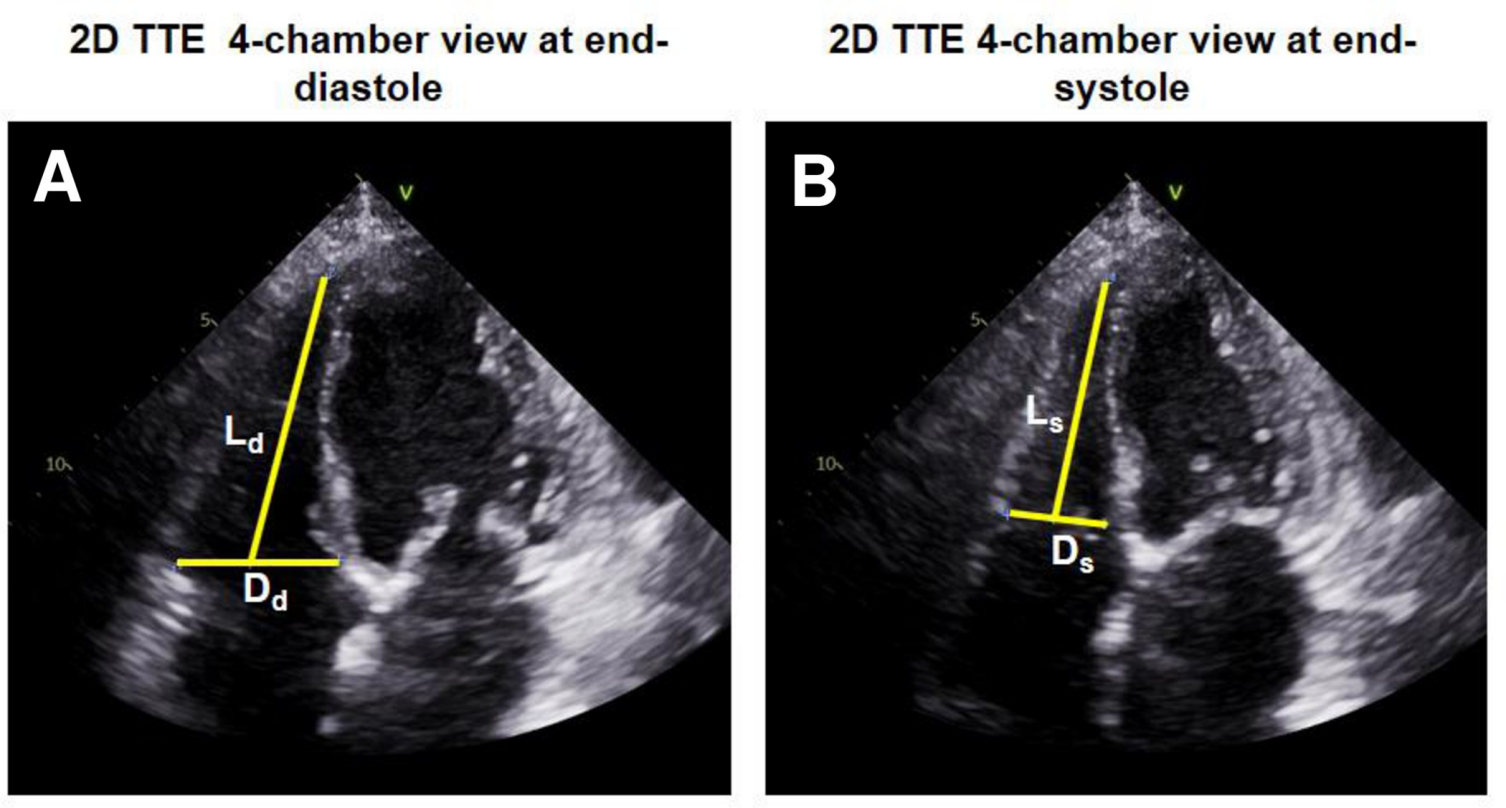
Linear dimensions measured in 2D TTE apical 4-chamber views for calculation of RV volumes and function by the proposed cone-pyramid formula (CPF).

Furthermore, the apical 4-chamber view was used for measurement of the RV basal diameter at the level of the tricuspid valve at end-diastole and end-systole (Dd and Ds, respectively) as well as for the measurement of baso-apical length from midpoint of RV base to RV apex at end-diastole and end-systole (Ld and Ls, respectively) (**Fig 1**). These two linear dimensions were used for calculations of end-diastolic volume (EDV) and end-systolic volume (ESV) of the RV using the following proposed cone-pyramid formula. RV ejection fraction (EF) was calculated as: RV EF = (RV EDV—RV ESV) / RV EDV * 100.

For 2D transthoracic echocardiography (TTE) calculation of RV volumes and function by the proposed cone-pyramid formula (CPF) basal diameter at the level of tricuspid valve (Dd and Ds, respectively) as well as baso-apical length from midpoint of RV basis to RV apex (Ld and Ls, respectively) were measured in apical 4-chamber views at end-diastole (A) and end-systole (B).

In 25 randomly selected patients TTE calculations of RV EDV, RV ESV and RV EF with the proposed cone-pyramid formula were repeated by the same observer at least one week after the first examination as well as by a second observer for assessment of intra- and interobserver agreement.

### Geometrical assumptions-based formula for determination of the RV

The RV volume was calculated based on the assumption of the RV shape being a truncated cone representing the whole RV space minus a narrow truncated rhomboid pyramid representing the portion of the LV protruding into this space. This will be mentioned as the cone-pyramid formula (CPF).

**Fig 2:**
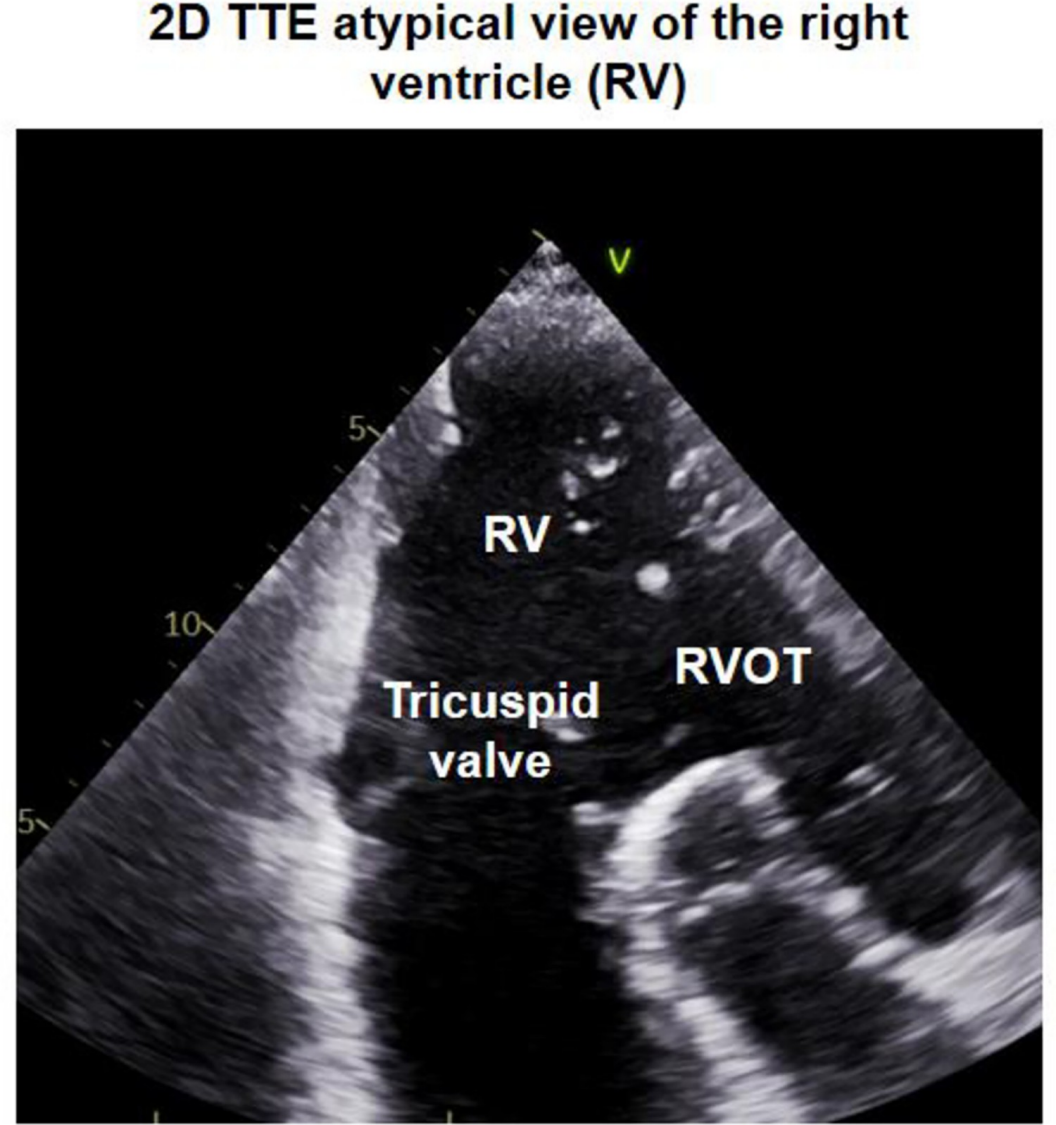
2D TTE atypical view of the RV displaying inflow and right ventricular outflow tract (RVOT) at once.

When looking in echocardiographic views different from the apical 4-chamber view the apex of the RV appears considerable wider than commonly assumed (**Fig 2**).

When adjusting a 2D TTE atypical right ventricular (RV) view different from the apical 4-chamber view displaying the inflow with the tricuspid valve and right ventricular outflow tract (RVOT) at once the apex of the RV appears considerable wider than commonly supposed.

According to that it was anticipated that the cross area of RV near the apex is 4/5 of the RV inflow area at the base. For further calculations, the radius (r) of the apical area was assumed to be 4/5 of the radius (R) of the basal RV inflow area at the level of the tricuspid valve: r = 4/5 R; D = 4/3 R.

**Fig 3:**
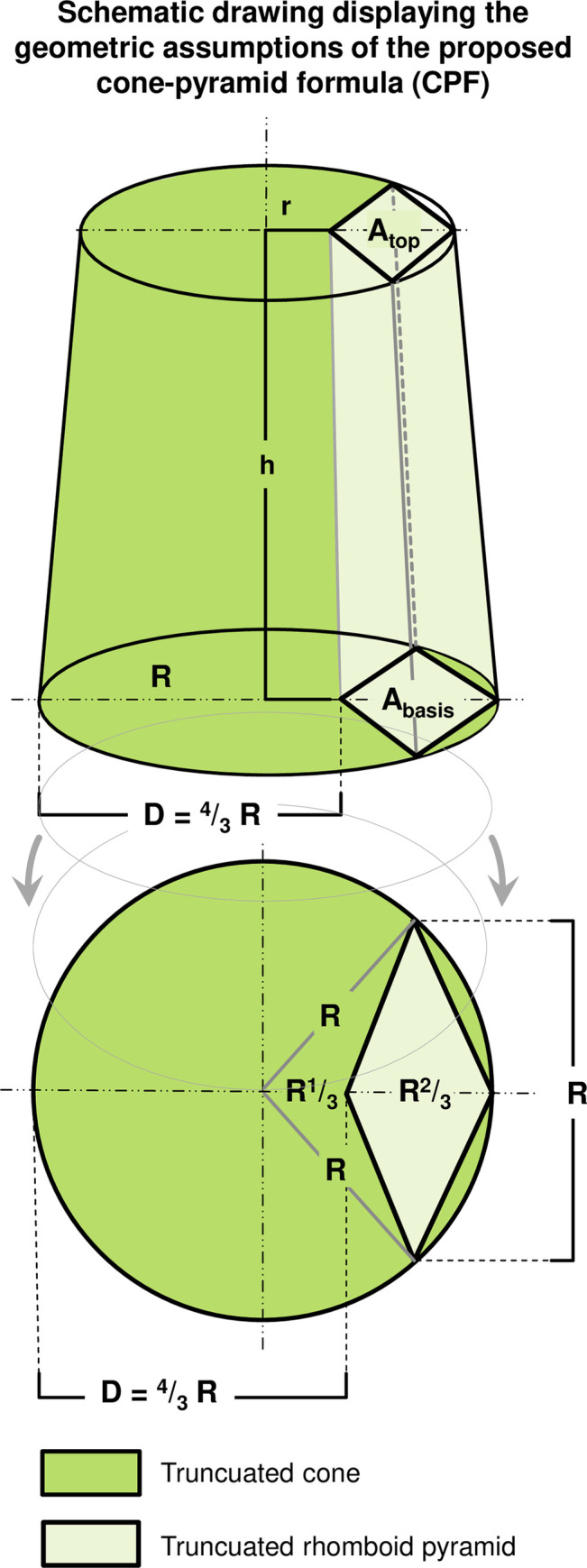
Schematic drawing displaying the geometric assumptions of the proposed cone-pyramid formula (CPF).

Geometrically truncated cone volume formula is: V_truncated cone_ = π/3 * h * (R^2^ + Rr + r^2^), where R is a radius of the basal area of the cone, and r is the top surface radius. It results: **V**_**truncated cone**_ = h * 1.44 * D^2^, where D represents the 2D TTE diameter of the RV basal diameter at the level of tricuspid valve in 4-chamber view and h represents the baso-apical length (L) from midpoint of RV basis to RV apex (**Fig 3, dark green**).

Right ventricular (RV) volumes and function were calculated based on the assumption of the RV shape being a truncated cone (darker green) representing the whole RV space minus a narrow truncated rhomboid pyramid (lighter green) representing the portion of the left ventricle protruding into this space. It was anticipated that the cross area of RV near the apex is 4/5 of the RV at the base. For further calculations, the radius (r) of the apical area was assumed to be 4/5 of the radius (R) of the basal RV area at the level of the tricuspid valve: r = 4/5 R. The basal diameter (D) was calculated as: D = 4/3 R. Hight (h) represents the baso-apical length (L) from mid part of the tricuspid valve to the RV apex. These calculations were made out of 2D transthoracic echocardiography (TTE) measurements in the apical 4-chamber view at end-diastole as well as at end-systole.

Accordingly, geometric formula of volume of truncated rhomboid pyramid is: V_truncated rhomboid pyramid_ = h/3 * (A_basis_ + A_top surface_ + √A_basis_ * A_top surface_), where h represents the baso-apical length (**L**) of the RV, A_basis_ the basal area and A_top_ the top surface area of the truncated rhomboid pyramid. Then, we assumed that 2/3 R belongs to the LV and 1/3 R belongs to the RV. That means D (diameter at level tricuspid valve) equals 4/3 R: A_basis_ = R^2^ * √2/4, A_top_ = r^2^ * √2/4, r = 4/5 R and D = 4/3 R. It results: **V**_**truncated rhomboid pyramid**_ = h* 0.23 * D^**2**^ (**Fig 3, light green**).

Finally, RV volume was calculated: V_RV_ = Vtruncated cone—V_truncated rhomboid pyramid_ = 1.44 * h * D^2^–0.23 * h * D^2^ resulting to **V**_**RV**_ = 1.21 * h * D^**2**^, where D represents the diameter of the RV basal diameter and h the baso-apical length (L) of the RV.

Consequently, RV end-diastolic and end-systolic volumes can be calculated by the developed CPF with 2D TTE measurements of RV basal diameters D at end-diastole and end-systole (Dd and Ds, respectively) and of baso-apical lengths at end-diastole and end-systole (Ld and Ls, respectively) in apical 4-chamber view (**Fig 1**).

### CMR protocol

The CMR studies were performed with a 1.5 Tesla whole-body magnetic resonance imaging scanner (Achieva, Philips Medical Systems, Best, The Netherlands) using a 32-channel cardiac coil. Analysis of the RV was achieved from the cine images using steady-state, free-precession (SSFP) sequences in 10–12 slices of 8-mm thickness, covering the whole RV from the base to the apex. As part of the CMR examination, the RV volumes were determined in end-diastole and in end-systole by contouring the endocardium on every short-axis slice. On the basis of these contours, RV EDV and RV ESV were calculated by the summation-of-discs method. RV EF was calculated as: RV EF = (RV EDV—RV ESV) / RV EDV * 100.

Patients were categorized as having enlarged RV, when RV EDV was >250 ml in men and >201 ml in women by CMR. Accordingly, impaired RV function was defined as RV EF <52% in men and <51% in women according to current CMR recommendations [4].

### Statistical analysis

Statistical analysis was performed with the MedCalc Statistical Software version 13.0 (MedCalc Software, Ostend, Belgium). Categorical variables were summarized as count (percentage) and continuous variables as mean±standard deviation. Scatter diagram with regression line and Bland-Altman plot with presentation of difference of arithmetic mean were used for display of agreement between measurements by TTE using the CPF and CMR. Correlations of different TTE variables with CMR measurements were determined by calculating the difference between the variables and using Spearman rank correlation coefficient. Additionally, correlations of TTE calculations and CMR measurements were evaluated separately differentiating between patients with enlarged RV and normal RV size as well as between patients with reduced and normal RV function as defined by CMR classification. Receiver-operating characteristics (ROC) curve analysis with calculation of area under the curve (AUC) and according 95%-confidence interval (CI) was performed for identification of patients with reduced RV function as defined by CMR classification. Differences between AUC´s were analyzed by pairwise comparison. Sensitivity and specificity of cut-off values with the highest Youden-index were presented. Intraclass correlation coefficient with 95%-CI was assessed for evaluation of intra- and interobserver agreement. P-values <0.05 were considered significant.

## Results

Patient characteristics and the different indications for RV assessment in all 129 patients are given in **Table 1**. According to CMR criteria RV was enlarged in 19 (15%) patients and RV function was reduced in 52 (40%) of all patients.

**Table 1:** Patient characteristics.

Variables	N = 129
Age, years	50±18
Male gender, n	89 (69%)
NYHA class	
I, n (%)	60 (47)
II, n (%)	21 (16)
III, n (%)	36 (28)
IV, n (%)	11 (9)
Indication for CMR, n	
Coronary artery disease	40 (31%)
Pericarditis/myocarditis	24 (19%)
Dilatative cardiomyopathy	14 (11%)
Congenital heart disease	12 (9%)
Valvular heart disease	11 (9%)
Cardiac arrhythmias	10 (8%)
Other	18 (14%)
Transthoracic echocardiography	
LV EF, %	49.5±13.8
TR	
no or mild, n (%)	103 (80)
moderate, n (%)	18 (14)
severe, n (%)	8 (6)
TAPSE, cm	2.0±0.6
SPAP, mmHg	29.6±13.2
RV Dd, cm	4.2±0.6
RV Ds, cm	3.4±0.6
RV Ld, cm	7.7±0.9
RV Ls, cm	6.3±1.1
RV diastolic area, cm^2^	22.3±5.9
RV systolic area, cm^2^	13.2±5.2
RV FAC, %	42.1±9.6
CMR	
RV EDV, ml	176.5±57.6
RV ESV, ml	89.7±47.6
RV EF, %	51.0±12.4

CMR: cardiovascular magnetic resonance, Dd: diastolic basal diameter of the right ventricle at the level of the tricuspid valve, Ds: systolic basal diameter of the right ventricle at the level of the tricuspid valve, EDV: end-diastolic volume, EF: ejection fraction, ESV: end-systolic volume, FAC: fractional area change, Ld: diastolic baso-apical length from mid part of the tricuspid valve to the right ventricular apex, Ls: systolic baso-apical length from mid part of the tricuspid valve to the right ventricular apex, LV-EF: left ventricular ejection fraction, NYHA: New York Heart Association, RV: right ventricular, SPAP: systolic pulmonary artery pressure, TAPSE: tricuspid annular plane systolic excursion, TR: tricuspid regurgitation.

In all 129 (100%) patients TTE measurements of linear dimensions in the apical 4-chamber views and calculation of RV EDV, RV ESV and RV EF using the proposed CPF was feasible.

### Comparison of RV size and RV function

**Fig 4:**
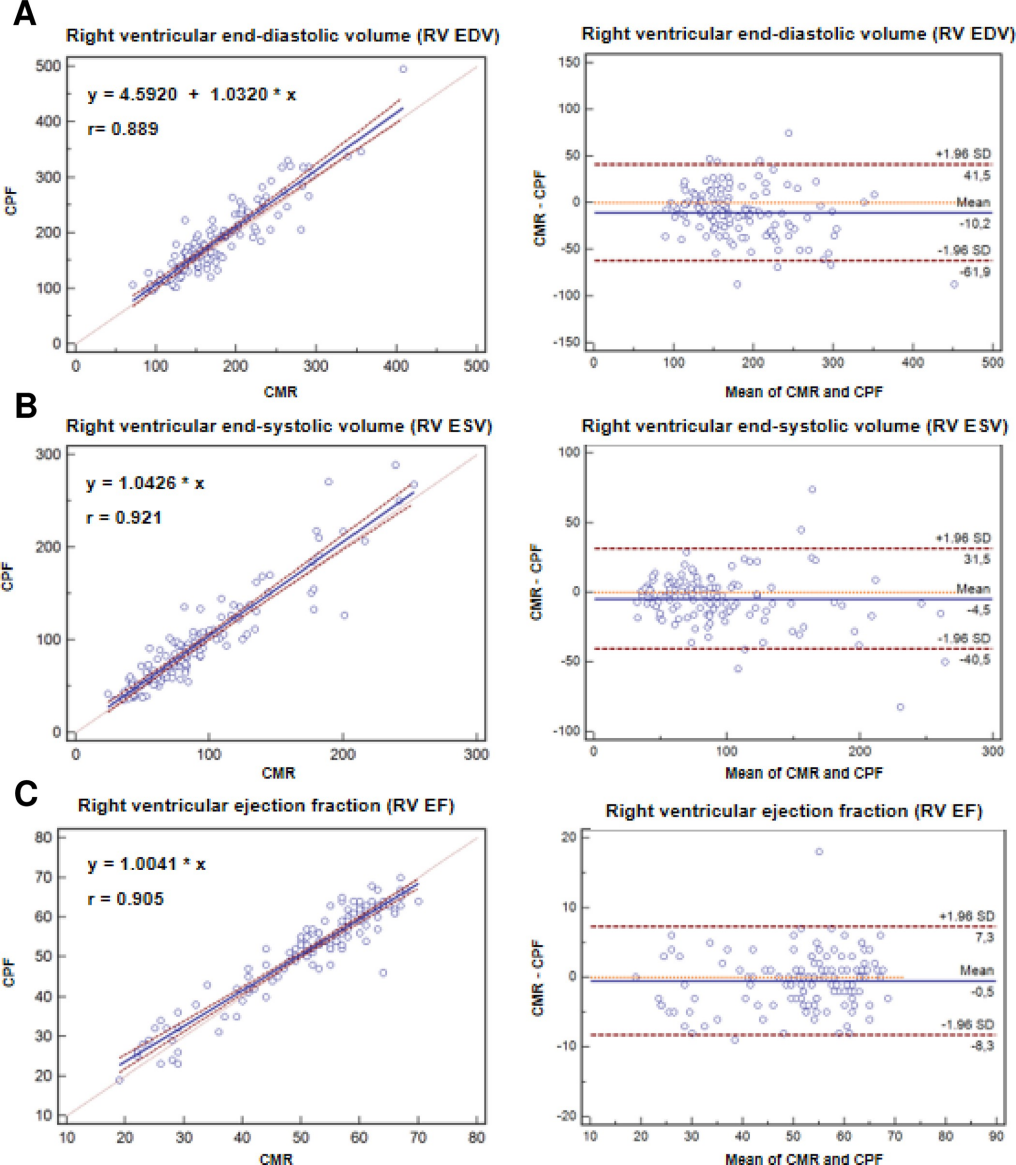
RV volumes and function by 2D TTE using the proposed cone-pyramid formula (CPF) and by CMR.

There was high agreement of RV EDV (ΔRV EDV = 10.2±26.4 ml, correlation coefficient r = 0.889) and RV ESV (ΔRV ESV = 4.5±18.4 ml, correlation coefficient r = 0.921) calculated by the proposed CPF and measured by CMR (**Table 2 and Fig 4A and 4B)**.

**Table 2:** Measurements of RV volumes and function by 2D TTE using the proposed cone-pyramid formula (CPF) and CMR. Calculations of RV end-diastolic volume (RV EDV), end-systolic volume (RV ESV) and ejection fraction (RV EF) by 2D TTE using the proposed cone-pyramid formula (CPF) compared to measurements by CMR.

	CMR	CPF	Difference between CMR and CPF	Correlation coefficient r
RV EDV, ml	176.5±57.6	186.7±65.0	10.2±26.4	0.889
RV ESV, ml	89.7±47.6	94.2±51.7	4.5±18.4	0.921
RV EF, %	51.0±12.4	51.6±11.8	0.5±4.0	0.905

CMR: cardiovascular magnetic resonance, CPF: cone-pyramid formula, EDV: end-diastolic volume, EF: ejection fraction, ESV: end-systolic volume, RV: right ventricular, TTE: transthoracic echocardiography.

Scatter diagrams with regression line and correlation coefficient (r) (left) and Bland-Altman plots with presentation of mean difference and standard deviations (SD) for display of agreement between calculations of RV end-diastolic volume (RV EDV), RV end-systolic volume (RV ESV) and RV ejection fraction (RV EF) by 2D TTE using the proposed cone-pyramid formula (CPF) and measurements by CMR (right).

These agreements of these CPF-based calculations with measurements obtained by CMR tended to be higher than correlations of CMR end-diastolic volume with conventional TTE parameters of RV size (RV basal diastolic diameter Dd to CMR RV EDV: correlation coefficient r = 0.778; RV diastolic area to CMR RV EDV: correlation coefficient r = 0.793) (**Table 3**).

**Table 3:** Correlation of 2D TTE and CMR measurements. Correlation of 2D TTE variables of RV size and CMR measurements of RV end-diastolic volume (RV EDV) and end-systolic volume (RV ESV) (above) as well as correlation of 2D TTE variables of RV function and RV ejection fraction (RV EF) measured by CMR (below).

RV size					
	CMR			CMR	
	RV EDV			RV ESV	
TTE variables	Correlation coefficient r		p-value	Correlation coefficient r	p-value
RV Dd	0.778		<0.0001	0.733	<0.0001
RV Ds	0.757		<0.0001	0.840	<0.0001
RV Ld	0.689		<0.0001	0.628	<0.0001
RV Ls	0.675		<0.0001	0.772	<0.0001
RV diastolic area	0.793		<0.0001	0.743	<0.0001
RV systolic area	0.727		<0.0001	0.782	<0.0001
RV EDV by CPF*	0.889		<0.0001	0.831	<0.0001
RV ESV by CPF*	0.821		<0.0001	0.921	<0.0001
RV function					
		**CMR**			
		**RV EF**			
**TTE**		Correlation		p-value	
**variables**		coefficient r			
TAPSE		0.592		<0.0001	
RV FAC		0.680		<0.0001	
RV EF by CPF*		0.905		<0.0001	

*Based on calculations by TTE measurements of two linear dimension with the proposed cone-pyramid formula (CPF)

CMR: cardiovascular magnetic resonance, CPF: cone-pyramid formula, Dd: diastolic basal diameter of the right ventricle at the level of the tricuspid valve, Ds: systolic basal diameter of the right ventricle at the level of the tricuspid valve, EDV: end-diastolic volume, EF: ejection fraction, ESV: end-systolic volume, FAC: fractional area change, Ld: diastolic baso-apical length from mid part of the tricuspid valve to the right ventricular apex, Ls: systolic baso-apical length from mid part of the tricuspid valve to the right ventricular apex, RV: right ventricular, TAPSE: tricuspid annular plane systolic excursion.

Furthermore, RV EF by the proposed CPF correlated well to measurements of RV EF by CMR (ΔRV EF = 0.5±4.0%, correlation coefficient r = 0.905) (**Table 2 and Fig 4C**). In contrast, there was only moderate correlation of conventional TTE measurements of RV function to RV EF measured by CMR (TAPSE to CMR RV EF: correlation coefficient r = 0.592; RV FAC to CMR RV EF: correlation coefficient r = 0.680) (**Table 3).**

### TTE calculations with the conus-pyramid formula in patients with enlarged RV and reduced RV function

TTE calculations of RV EDV and RV ESV by the proposed CPF correlated similarly well to CMR measurements in patients with enlarged RV (n = 19; ΔRV EDV = 9.1±40.1 ml, correlation coefficient r = 0.743; ΔRV ESV = 3.5±34.7 ml, correlation coefficient r = 0.849) as in patients with normal RV size (n = 110; ΔRV EDV = 10.4±23.5 ml, correlation coefficient r = 0.843; ΔRV ESV = 4.7±14.1 ml, correlation coefficient r = 0.892). There was similar agreement on RV EF by the CPF and by CMR measurement in patients with reduced RV function as defined by CMR (n = 52; ΔRF EF = 1.8±3.6%, correlation coefficient r = 0.901) as in patients with normal RV function (n = 77; ΔRV EF = 0.4±4.0%, correlation coefficient r = 0.873).

### Identification of patients with reduced RV function

**Fig 5:**
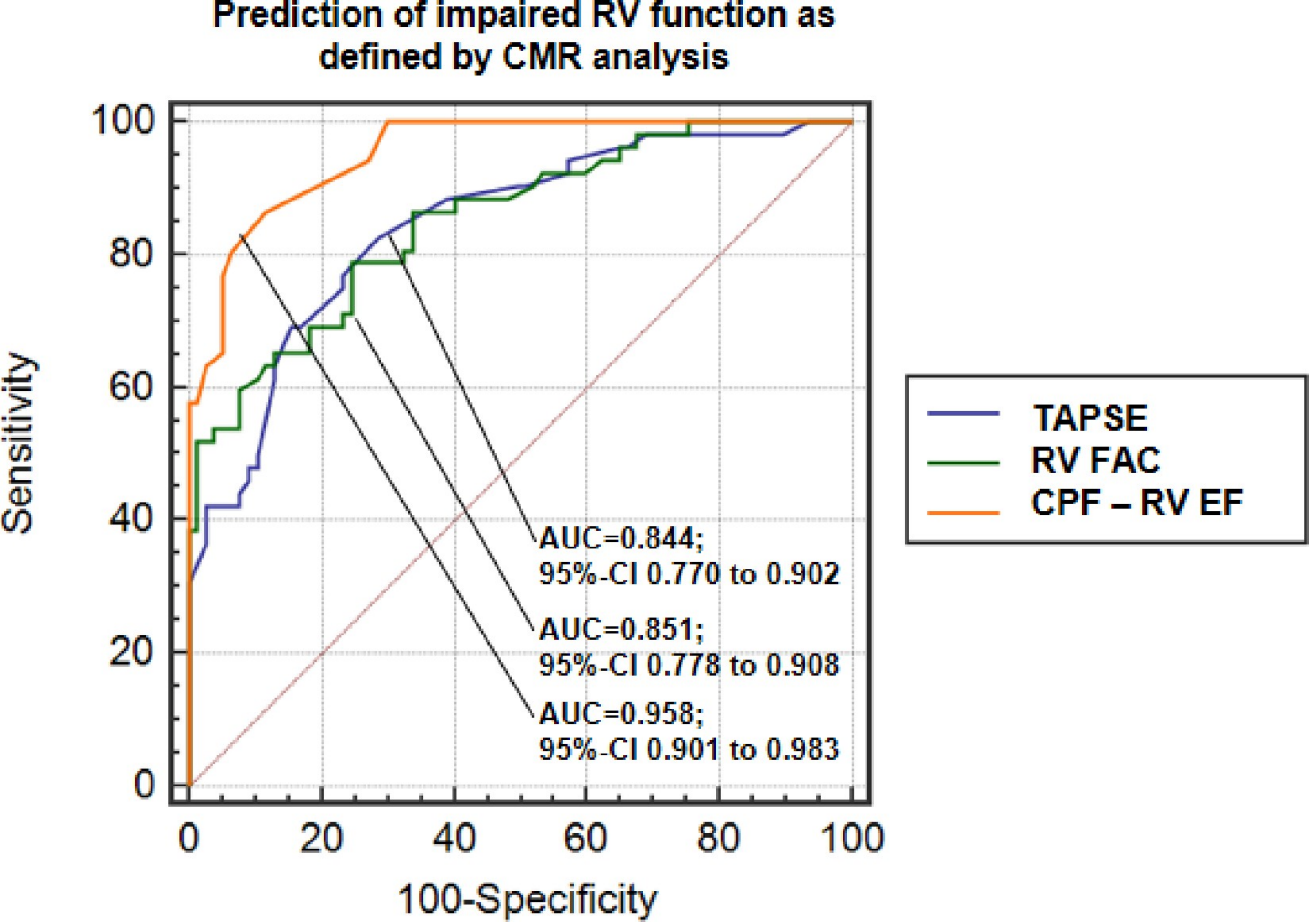
Identification of patients with reduced RV function.

Patients with reduced RV function (n = 52) as classified by CMR criteria were identified with high accuracy using the proposed CPF (AUC = 0.958, 95%-CI 0.901 to 0.983; cut-off value: ≤52%, sensitivity = 86.5%, specificity = 88.3%). Accuracy for identification of patients with reduced RV EF using the CPF was higher than by TAPSE and RV FAC (TAPSE: AUC = 0.844, 95%-CI 0.770 to 0.902; cut-off value: ≤2.0 cm, sensitivity = 82.7%, specificity = 71.4%; RV FAC: AUC = 0.851, 95%-CI 0.778 to 0.908; cut-off value: ≤41.8%, sensitivity = 78.9%, specificity = 75.3%) as conventional TTE measurements of RV function (differences between AUC´s: p = 0.8694 for TAPSE vs. RV FAC; p = 0.0018 for TAPSE vs. CPF RV EF; and p = 0.0011 for RV FAC vs. CPF RV EF) (**Fig 5**).

ROC analysis of 2D TTE variables of tricuspid annular plane systolic excursion (TAPSE, blue line), right ventricular fractional area change (RV FAC, green line) and RV ejection fraction (RV EF, orange line) using the proposed cone-pyramid formula (CPF) for prediction of impaired RV function as defined by CMR classification.

### Intraobserver and interobserver agreement

Agreement between repeated measurements in 25 patients by the same observer with TTE calculations using the proposed CPF of RV EDV (ΔRV EDV = -3.9±30.0 ml, ICC = 0.8571), RV ESV (ΔRV ESV = 1.5±21.3 ml, ICC = 0.9235) and RV EF (ΔRV EF = -1.4±5.0%, ICC = 0.9322) was high with only little difference between the measured values. Accordingly, agreement between repeated measurements by a second observer with calculations of RV EDV (ΔRV EDV = -8.6±24.8 ml, ICC = 0.8994), RV ESV (ΔRV EDV = -9.6±18.3 ml, ICC = 0.8999) and RV EF (ΔRV EF = 1.1±9.2%, ICC = 0.7233) using the CPF was also high with reasonable differences between the measured values (**Table 4**).

**Table 4:** Intra- and interobserver agreement of measurements of RV volumes and function by 2D TTE using the proposed cone-pyramid formula (CPF). Intra- and interobserver agreement of 2D TTE calculations using the proposed cone-pyramid formula (CPF) of RV end-diastolic volume (RV EDV), RV end-systolic volume (RV ESV) and RV ejection fraction (RV EF) in 25 randomly selected patients by re-measurements of the initial observer 1 and by a second observer 2.

	Observer 1			Observer 2	
	CPF Measurement 1	CPF Measurement 2	Intraobserver ICC with 95%-CI	CPF Observer 2	Interobserver ICC with 95%-CI
RV EDV, ml	181.6±50.2	177.8±60.2	0.8571 [0.7040–0.9343]	173.1±63.2	0.8994 [0.7827–0.9546]
RV ESV, ml	98.1±49.3	99.6±57.6	0.9235 [0.8346–0.9656]	88.5±40.2	0.8999 [0.7492–0.9578]
RV EF, %	47.7±13.5	46.2±14.2	0.9322 [0.8531–0.9695]	48.8±11.4	0.7233 [0.4664–0.8678]

CI: confidence interval, CPF: cone-pyramid formula, EDV: end-diastolic volume, EF: ejection fraction, ESV: end-systolic volume, ICC: intraclass correlation coefficient, RV: right ventricular, TTE: transthoracic echocardiography.

## Discussion

The major findings of this study are: (1) calculations of RV volumes and function by 2D TTE with the proposed CPF based on only measurements of two linear dimensions are feasible with high agreement with CMR measurements as the reference method, (2) calculations of RV volumes and function are reproducible with high intra- and interobserver agreement, (3) calculations of RV EDV, RV ESV and RV EF with the CPF could be performed in patients with normal RV size and function as well as in patients with enlarged RV and impaired RV function, and (4) accuracy of RV EF analysis using the CPF for detection of patients with reduced RV function as defined by CMR classification was higher than those using conventional TTE parameters for RV function analysis.

### Geometry based echocardiographic models for RV volumetric analysis

The function of right heart has an important impact on morbidity and prognosis of patients with terminal heart failure, pulmonary hypertension and acute myocardial infarction. The difficulty of estimating RV volumes and function with 2D echocardiography is well known, predominantly due to the complex anatomy of the RV with its crescent geometry when viewed in a transverse section of the heart [1]. The longitudinal function of the RV (as e.g. assessed by TAPSE) may not express the true function of the RV as the axial function also importantly contributes to its function. Therefore, it is important to determine longitudinal and axial functions in order to properly assess the systolic function of the right ventricle. The proposed CPF evaluated in this study allows a more accurate quantification of RV function by combining both components of function, the longitudinal (RV length shortening in systole) and the axial function component (RV width reduction in systole).

In previous studies, different echocardiographic approaches of calculating RV volume have been described. Area-length based methods, initially adopted for biplane angiography, use approximations of the crescentic like RV geometry, most commonly based on modified pyramidal, ellipsoid or tapering models [2]. A study by Aebischer et al. showed high correlation between 2D TTE derived RV EDV in five models and CMR assessment as the reference method. Thereby, two crescentic models slightly underestimated RV volume (ΔRV EDV = -10.2 ml, correlation coefficient r = 0.99; ΔRV EDV = -10.5 ml, correlation coefficient r = 0.94, respectively), whereas a tapering (ΔRV EDV = -50.1 ml, correlation coefficient r = 0.94) and a pyramidal model (ΔRV EDV = -89.7 ml, correlation coefficient r = 0.94) underestimated it in a much larger proportion and an ellipsoid model presented with slight overestimation (ΔRV EDV = 1.7 ml, correlation coefficient r = 0.90). However, all evaluated models required analysis of a combination of measurements obtained from a short axis and an apical view [5]. Due to their complexity, the use of these 2D TTE based methods for assessment of RV volumes is not recommended in the current guidelines of chamber quantification [3]. To overcome this problem, we evaluated a newly developed CPF in our study which is based only on easy to perform 2D TTE measurements of two linear dimensions in apical 4-chamber view. In our investigation we found results (ΔRV EDV = 10.2 ml, correlation coefficient r = 0.889) that were comparable to the two presented crescentic models and the ellipsoid model presented by Aebischer et al. without the need of complex measurements in several views [5].

### Alternative methods of RV assessment

Multiple studies have demonstrated the clinical utility and value of TAPSE and RV FAC and the use of these echocardiographic parameters is recommended by the current guidelines for evaluation of RV function [3]. TAPSE is an echocardiographic parameter easily obtainable with high interobserver agreement (ICC = 0.95), but it is angle dependent and this parameter is only partially representative of RV global function [3, 6]. RV FAC is also an established echocardiographic parameter with prognostic value, but this variable has only fair inter-observer reproducibility (ICC between 0.62 to 0.82) [3, 6, 7]. In our study RV EF calculated by 2D TTE with the proposed CPF was superior in identification of patients with reduced RV EF (AUC = 0.958) as defined by CMR classification compared to TAPSE (AUC = 0.844, p = 0.0018) and RV FAC (AUC = 0.851, p = 0.0011). Our findings of accuracy of TAPSE (AUC = 0.84) and RV FAC (AUC = 0.85) are on the same level as previous investigations of Tong et al. for identification of patients with impaired RV function as defined by CMR analysis [8]. In contrast to TAPSE and RV FAC which are not exactly comparable parameters of RV function, we calculated RV EF values by 2D TTE using the CPF which are directly comparable to the values assessed by CMR measurements (ΔRV EF = 0.5±4.0%, correlation coefficient r = 0.905).

Tissue Doppler and speckle-tracking based methods were not investigated in our study. Analogous to TAPSE measurement of tricuspid lateral annular systolic velocity (S´) provides information about longitudinal RV function. Current guidelines assume strain imaging by speckle-tracking echocardiography to be feasible for RV function analysis. Although its regular use was not recommended in the current recommendations for cardiac chamber quantification due to a lack of accepted reference ranges, recent papers investigated these values, so that speckle-tracking echocardiography is increasingly used in the clinical routine [3, 9].

3D assessment of RV volumes and function has been suggested to overcome the limitations of 2D TTE. But even in well performed 3D echocardiographic investigations like in the study of Leibundgut et al. (ΔRV EDV = 10.2 ml to CMR, correlation coefficient r = 0.84) and Namisaki et al. using manual editing as well as fully automated RV quantification (ΔRV EDV between 2 ml to 10 ml to CMR, correlation coefficients between 0.86 to 0.88) 3D TTE analysis was only possible in 92% of the patients [10, 11]. Due to technical challenges, particularly in patients with imperfect image quality and in significantly dilated or dysfunctional ventricles, 3D TTE evaluation of the RV is recommend only in laboratories with 3D echocardiographic experience [2, 3]. In contrast, in our study RV volume and function analysis using the CPF was not only accurate in all patients with normal RV size and function, but also in all patients with enlarged RV and with reduced RV EF.

CMR is the accepted gold standard for quantification of RV volumes and RV EF. However, CMR has limitations in patients with metallic implants, obesity, arrhythmias, and claustrophobia. Furthermore, there is limited availability of CMR and high costs have to be considered [4]. The 2D TTE CPF method presented in our study may provide a simple and practical alternative with acceptable accuracy when RV assessment is indicated. It should be taken into account, that we have compared RV volumes and EF in 2D TTE with those measured in CMR. This may explain a better agreement of RV volumes and EF calculated by CPF and measured by CMR than of conventional TTE parameters for RV quantification like TAPSE or RV FAC.

### Limitations

The main limitation is that this study was single center and based on retrospective analyses. Furthermore, no detailed clinical characterization was performed and no prognostic data have been investigated to evaluate the clinical impact of the new 2D echocardiographic method of RV assessment with the proposed CPF on patient´s outcome.

## Conclusions

Calculations of RV volumes and function by 2D TTE with the proposed CPF based only on measurements of two linear dimensions obtained in the apical 4-chamber view were feasible in all patients with high concordance to CMR measurements. These calculations were performed with high accuracy in both patients with normal RV size and function as well as in patients with enlarged RV and impaired RV function. The CPF was more accurate in identification patients with reduced RV function as defined by CMR compared to conventional echocardiographic parameters.
